# Plasma Fibrinogen Predicts Response to Immune Checkpoint Inhibitor by Inflammatory Tumor Microenvironment in Esophageal Cancer

**DOI:** 10.1002/cam4.71548

**Published:** 2026-01-18

**Authors:** Keiso Ho, Satoru Matsuda, Eisuke Booka, Wataru Soneda, Jun Okui, Shota Hoshino, Masashi Takeuchi, Kazumasa Fukuda, Sara Horie, Yuki Saito, Yasunori Kogure, Hirofumi Kawakubo, Kensuke Hara, Hajime Okita, Keisuke Kataoka, Shigeki Sekine, Hiroya Takeuchi, Yuko Kitagawa

**Affiliations:** ^1^ Department of Surgery Keio University School of Medicine Tokyo Japan; ^2^ Department of Surgery Hamamatsu University School of Medicine Hamamatsu Japan; ^3^ Division of Gastroenterology and Hepatology, Department of Internal Medicine Keio University School of Medicine Tokyo Japan; ^4^ Division of Molecular Oncology National Cancer Center Research Institute Tokyo Japan; ^5^ Department of Pathology Keio University School of Medicine Tokyo Japan; ^6^ Division of Diagnostic Pathology Keio University School of Medicine Tokyo Japan; ^7^ Division of Hematology, Department of Medicine Keio University School of Medicine Tokyo Japan

**Keywords:** biomarkers for immunotherapy response, esophageal cancer, fibrinogen, immune checkpoint inhibitor, tumor‐associated neutrophil

## Abstract

**Background:**

Plasma fibrinogen (FNG) is a prognostic marker in esophageal squamous cell carcinoma (ESCC). However, its predictive value for immune checkpoint inhibitor (ICI) efficacy and the underlying mechanisms remain unclear. This study aimed to evaluate the clinical significance of plasma FNG levels in ICI‐treated ESCC patients and investigate its association with tumor‐associated neutrophils (TANs) and genomic alterations.

**Methods:**

A retrospective, multicenter analysis of 167 ESCC patients treated with ICIs was performed. TANs were quantified via immunohistochemistry using CD11b and CD66b staining, and PD‐L1 expression was assessed using the tumor proportion score (TPS). Whole‐exome and RNA sequencing were conducted to analyze genomic and transcriptomic profiles.

**Results:**

Elevated plasma FNG levels correlated with lower ICI response rates and decreased survival. In first‐line treatment, chemo‐ICI therapy demonstrated superior efficacy compared to dual‐ICI therapy in high‐FNG patients, while the reverse trend was observed in low‐FNG patients. High‐FNG tumors showed increased TAN infiltration, independent of PD‐L1 expression. RNA sequencing revealed enrichment of neutrophil activation and extravasation pathways in high‐FNG tumors.

**Conclusions:**

Elevated plasma FNG levels predict poor prognosis and reduced ICI efficacy in ESCC. They may be potential biomarkers for first‐line ICI‐based therapy and correlate with TAN infiltration. Further validation and mechanistic investigations are warranted.

**Abbreviations**
CF: cisplatin and 5‐fluorouracil
CNAs: copy number alterations
CT: computed tomography
DCF: docetaxel, cisplatin, and 5‐fluorouracil
ESCC: esophageal squamous cell carcinoma
FNG: fibrinogen
FU: fluorouracil
ICI: immune checkpoint inhibitor
irAE: immune‐related adverse events
OS: overall survival
ssGSEA: single‐sample gene set enrichment analysis
TAN: tumor‐associated neutrophil
TME: tumor microenvironment
TPS: tumor proportion score
UICC: the Union for International Cancer Control
VAF: variant allele frequency
WES: whole‐exome sequencing

## 1 Introduction

Esophageal squamous cell carcinoma (ESCC) represents the seventh leading cause of cancer‐related mortality worldwide and is characterized by its propensity to induce lymph node metastasis, even in the incipient phases of the disease [1, 2, 3]. While multidisciplinary therapeutic strategies, encompassing surgical intervention, chemotherapy, and radiotherapy, have been developed to enhance patient outcomes, the accurate assessment of recurrence risk and the identification of supplementary treatment modalities for high‐risk individuals remain significant clinical challenges. Consequently, there exists a pressing need for the development of simple and reliable prognostic biomarkers [4, 5].

Among the diverse prognostic markers employed in esophageal cancer, plasma fibrinogen (FNG) levels are acknowledged for their ease of measurement and high degree of accuracy. Extensive research has substantiated the clinical significance of plasma FNG levels in predicting the prognosis of a spectrum of malignancies, encompassing esophageal, colorectal, lung, and ovarian cancers [6, 7, 8, 9, 10, 11]. Our research group has previously documented the utility of preoperative FNG levels and the fibrinogen‐albumin score in forecasting long‐term outcomes in esophageal cancer [12, 13, 14, 15, 16]. Although elevated plasma FNG levels have been consistently identified as an adverse prognostic factor in ESCC in previous investigations [15, 16, 17, 18, 19], the precise mechanisms underlying this association remain to be fully elucidated.

Immune checkpoint inhibitors (ICIs) have become a standard treatment for metastatic ESCC, but the risk of immune‐related adverse events (irAEs) highlights the critical need for predictive biomarkers. With chemo‐ and dual‐ICI therapies now being standard first‐line options for advanced ESCC, following the KEYNOTE‐590 and CheckMate 648 trials [20, 21], biomarkers are urgently needed to guide treatment decisions due to varying tumor shrinkage, costs, and irAE rates. We previously reported FNG as a potential ICI efficacy predictor in a cohort of 50 ESCC patients undergoing ICI monotherapy [22]. However, further research is required to validate these findings in larger cohorts and to include data on chemo‐ and dual‐ICI therapies. Moreover, the underlying mechanisms linking FNG levels to ICI efficacy and tumor progression remain largely uncharacterized.

This study validated serum FNG levels as a predictive biomarker for ICI efficacy in an expanded multicenter cohort. We further investigated FNG's value in guiding the stratified use of chemo‐ and dual‐ICI therapies, current first‐line treatments. Hypothesizing that primary tumor molecular profiles differ between high‐ and low‐FNG ESCC patients, we performed a comprehensive analysis of primary tumor samples to elucidate the relationship between FNG levels and molecular characteristics of ESCC.

## 2 Materials and Methods

### 2.1 Study Design and Patient Selection

This multicenter retrospective study was conducted by Keio University and Hamamatsu University School of Medicine. The study population comprised patients with unresectable advanced or recurrent thoracic ESCC who underwent treatment with ICIs, such as nivolumab, pembrolizumab, or ipilimumab, between January 2017 and March 2024 at either of the participating institutions. In this study, we included patients with unresectable advanced or recurrent ESCC who were treated with standard regimens, including chemo‐ICI or dual‐ICI therapy, in accordance with the Japanese Esophageal Cancer Practice Guidelines (2022 edition) [23], which are based on the results of the KEYNOTE‐590 and CheckMate 648 trials [20, 21]. Tumors located in the cervical esophagus, as classified according to the Japanese Classification of Esophageal Cancer [24], were excluded. Additionally, patients with a history of ICI therapy for other malignancies were excluded. Patient characteristics, clinicopathological features, laboratory data, and long‐term outcomes were analyzed retrospectively. Tumor staging was conducted following the 8th edition of the TNM Classification published by the Union for International Cancer Control (UICC) [25]. Ethical approval for this study was granted by the Ethics Committee of Keio University (Registration No: 20231201) and Hamamatsu University School of Medicine (Registration No: 91‐244). This study was retrospective in design, and because no previous study has specifically investigated survival rates in patients with unresectable advanced or recurrent ESCC presenting with elevated pretreatment FNG levels, it was difficult to predict the hazard ratio between the high‐ and low‐FNG groups a priori. Therefore, a target sample size was not set based on statistical power. Alternatively, statistical power was retrospectively calculated according to the log‐rank test using Schoenfeld's method on the observed data to demonstrate the reliability of our causal inference. A *p*‐value of < 0.05 was considered statistically significant.

### 2.2 Treatment and Follow‐Up

For the treatment of unresectable advanced or recurrent esophageal cancer, cisplatin, 5‐fluorouracil (FU), and pembrolizumab (CF + Pembrolizumab); cisplatin, 5‐FU, and nivolumab (CF + Nivolumab); and nivolumab plus ipilimumab have been established as standard first‐line treatment regimens since 2020 [20, 21]. In this study, CF + pembrolizumab and CF + Nivolumab were designated as chemo‐ICI therapy, while nivolumab plus ipilimumab was categorized as dual‐ICI therapy. Patients who received alternative ICI therapies, such as nivolumab monotherapy, either as second‐line treatment or for other clinical indications, were also incorporated into the analysis.

Nivolumab monotherapy was administered in accordance with the ATTRACTION‐3 trial protocol [26]. In cases of resectable advanced ESCC, transthoracic esophagectomy, utilizing either open or minimally invasive surgical techniques, was performed, followed by gastric tube reconstruction via the posterior mediastinal route. Bilateral recurrent laryngeal nerve dissection and abdominal lymphadenectomy, encompassing lymph nodes surrounding the stomach, lesser curvature, and left gastric artery, were routinely performed [27]. For tumors located within the upper or middle thoracic esophagus, supraclavicular lymph node dissection was additionally performed.

In accordance with previous investigations [28], both institutions initially implemented a preoperative treatment regimen consisting of cisplatin and 5‐fluorouracil (CF) administered every 3 weeks for two cycles. Subsequently, upon the demonstration of the efficacy of the docetaxel, cisplatin, and 5‐FU (DCF) regimen in the JCOG1109 trial [29], we transitioned to utilizing DCF administered every 3 weeks for three cycles as the standard preoperative chemotherapy regimen, commencing in January 2022.

Patients received follow‐up computed tomography (CT) scans at 2–3 month intervals. Treatment strategies were determined through multidisciplinary oncology conferences, which included surgical and medical oncologists, radiologists, and radiation oncologists. Overall survival (OS) was defined as the time elapsed from the initiation of any ICI‐based regimen to death from any cause. Follow‐up was conducted until disease recurrence or death, concluding in October 2024. Treatment response was assessed according to the Revised RECIST Guideline version 1.1 [30].

### 2.3 Measurement of Plasma FNG Levels

Preoperative plasma FNG levels were measured from early morning blood samples, obtained prior to breakfast, and collected within 7 days preceding surgical intervention. For patients with unresectable or recurrent disease, plasma FNG levels were assessed within 1 month prior to the commencement of treatment. FNG assays were performed using the Clauss method, which is based on the thrombin clotting time [31], utilizing Datafai Fibrinogen (Sysmex Corporation, Kobe, Japan) on a CA7000 analyzer (Sysmex Corporation).

For unresectable advanced and recurrent ESCC, pretreatment FNG levels were stratified into two groups using a 390 mg/dL cutoff. This cutoff, maximizing the Youden index on the ROC curve, ensures optimal sensitivity and specificity, consistent with prior studies [32, 33]. To directly assess tumor burden, we compared the number of metastatic organs between groups. Metastatic organs were defined as the total count of metastatic involvement across the following sites: the primary or locally recurrent tumor (0 or 1), bone metastasis (0 or 1), number of lung metastases, number of liver metastases, presence of regional and extra‐regional lymph node metastases in the cervical, thoracic, and abdominal regions (each scored as 0 or 1), brain metastasis (0 or 1), peritoneal dissemination (0 or 1), and other organ metastases including muscle or skin involvement (0 or 1).

Further, in the analysis of surgical specimens from patients with resectable ESCC who underwent tissue staining and genomic analysis, hyperfibrinogenemia was defined as an FNG level exceeding 350 mg/dL, consistent with our previously published report [15]. This threshold is concordant with the upper limit of the normal reference range established at our institution.

### 2.4 CD11b and CD66b Immunohistochemical Staining

Formalin‐fixed tumor tissues (24 h) were paraffin‐embedded and sectioned into 4‐μm slices. Deparaffinization and antigen retrieval were performed using Tris (hydroxymethyl) aminomethane (pH 9.0, NACALAI TESQUE, INC, Kyoto, Japan) at 121°C for 5 min, followed by nonspecific binding reduction with BLOXALL (Vector Laboratories, INC., Newark, CA, USA) for 10 min. Sections were incubated overnight at 4°C with primary antibodies: CD11b (1:4000; clone EPR1344; Abcam, Cambridge, UK) or CD66b (1:100; polyclonal; Abcam). Secondary antibodies (EnVision + System‐HRP Labeled Polymer, Dako, Agilent Technologies, INC., Santa Clara, CA, USA) were applied, and visualization was achieved using the Peroxidase Stain DAB Kit (NACALAI TESQUE, INC.), with hematoxylin counterstaining. A pathologist confirmed staining validity. Five random fields (≥ 50% tumor content) were selected, and stained cells were counted at 400× magnification. Pathological evaluation was conducted single‐blind.

### 2.5 PD‐L1 Immunohistochemical Staining

The PD‐L1 immunostaining procedure was outsourced to Morphotechnology, a specialized immunostaining facility, and performed with the PD‐L1 IHC 28‐8 kit (Dako Code: SK005), following the manufacturer's guidelines. Formalin‐fixed tumor tissues (24 h) were paraffin‐embedded and sectioned into 4‐μm slices. Deparaffinization and antigen retrieval were then performed using Target Retrieval Solution (pH 9.0, Dako Code: S1699) at 97°C for 30 min.

Incubation of sections with a primary PD‐L1 antibody (1:250; clone 28‐8; Abcam) was performed at 20°C for 60 min. Visualization was then carried out using the Rabbit Linker reagent and EnVision (Dako), followed by hematoxylin counterstaining.

A pathologist confirmed the validity of the PD‐L1 staining. PD‐L1 expression was evaluated using the tumor proportion score (TPS), adhering to the CheckMate 648 trial protocol [20]. The pathological evaluation was performed in a single‐blind manner.

### 2.6 Sample Preparation for Whole‐Exome and RNA Sequencing

During surgery, tissue samples were collected from primary esophageal cancer lesions and immediately frozen at −80°C. Tumor genomic DNA and RNA extraction were performed using the QIAamp DNA Mini kit (QIAGEN, Hilden, Germany) and RNeasy Mini Kit (QIAGEN), respectively. For matched normal genomic DNA, formalin‐fixed paraffin‐embedded normal esophageal tissues, also collected during surgery, were used with the GeneRead DNA FFPE Kit (QIAGEN).

### 2.7 Whole‐Exome Sequencing

The SureSelect Human All Exon V6 capture kit (Agilent) was used to prepare libraries for whole‐exome sequencing (WES). Sequencing was then performed on the NovaSeq6000 platform (Illumina, San Diego, CA, USA) at Macrogen (Seoul, South Korea), yielding 150‐bp paired‐end reads.

The Genomon pipeline (https://github.com/Genomon‐Project) version 2.6.3, with minor modifications previously described [34, 35], was used for sequence alignment and mutation calling. Reads were aligned to the hs37d5 human reference genome. Putative somatic mutations were selected based on: (i) Fisher's exact *p* < 0.1; (ii) EBCall *p* < 1 × 10^−5^ [36]; (iii) ≥ 4 variant reads in tumor; (iv) tumor variant allele frequency (VAF) > 0.05; and (v) germline VAF < 0.02. These were filtered by removing: (i) synonymous single nucleotide variants, (ii) variants in repetitive regions; and (iii) variants in unidirectional reads. Candidate mutations were then filtered further, unless found ≥ 10 times in COSMIC version 70, by excluding known germline variants (i) from dbSNP build 131, or (ii) with frequency ≥ 0.0001 in 1000 Genomes (released in October 2014), National Heart, Lung, and Blood Institute Exome Sequencing 6500, the Human Genome Variation Database version 2.00, Tohoku Medical Megabank Organization 3.5KJPNv2, or Exome Aggregation Consortium r0.3.1. For one sample without normal control, Fisher's exact and germline VAF criteria were omitted, but the same filtering was applied. Somatic mutations in 24 driver genes reported previously [37] were considered driver mutations.

CNACS, as previously described [34, 35], was used to detect copy number alterations (CNAs). These were then filtered, removing those (i) with fewer than three probes and (ii) with a total copy number between 1.8 and 2.2. Both focal (shorter than half a chromosome arm) and arm‐level CNAs underwent manual revision. Driver CNAs were defined as arm‐level and focal CNAs reported in a previous study [38].

### 2.8 RNA Sequencing

Libraries for RNA sequencing were generated utilizing the TruSeq Stranded mRNA (Illumina) and subsequently sequenced on the NovaSeq6000 platform (Illumina) at Macrogen, yielding 100 base pair paired‐end data. Mapping was conducted using Genomon version 2.6.3, employing the same reference sequence as the WES analysis. Ensembl gene expression was quantified using featureCounts [39]. Single‐sample gene set enrichment analysis (ssGSEA) was conducted via ssGSEA2.0 R package, which enabled the calculation of scores for two MSigDB gene sets: regulation of neutrophil activation (M25086, GOBP_REGULATION_OF_NEUTROPHIL_ACTIVATION) and neutrophil extravasation (MM9966, GOBP_NEUTROPHIL_EXTRAVASATION).

### 2.9 Statistical Analysis

For the univariate analysis of background characteristics and predictors of histological response, chi‐squared tests were applied to categorical variables, while Mann–Whitney *U* tests were utilized for continuous variables to determine differences between the two groups. Prognostic analyses were conducted using the Kaplan–Meier method and log‐rank tests. Multivariate survival analysis was conducted using cox regression model. All statistical analyses were performed using Stata/IC 17 for Mac (StataCorp, College Station, TX, USA) and R version 3.1.2 (R Foundation Statistical Computing, Vienna, Austria). A significance level of *p* < 0.05 was established for all statistical tests.

## 3 Results

### 3.1 Impact of FNG on ICI Response and Prognosis

Between January 2017 and March 2024, we reviewed 167 patients with previously treated unresectable progression or recurrent ESCC who received ICI therapy at Keio University and Hamamatsu University School of Medicine. Of these, 154 patients had available pre‐ICI serum FNG data: 95 in the high‐FNG group and 59 in the low‐FNG group. There was no significant difference in patient background between the two institutions. In addition, FNG levels were not significantly different, with mean ± SD values of 426.2 ± 12.1 at Keio University and 454.5 ± 18.8 at Hamamatsu University. The cohort consisted of a balanced number of patients with recurrent and metastatic disease, and approximately half received ICIs as first‐line therapy (Table 1). To directly assess tumor burden, we compared the number of metastatic organs between groups. No significant differences were observed between the high‐ and low‐FNG groups in the overall cohort of 154 patients (3.7 ± 3.4 vs. 3.3 ± 3.1, *p* = 0.246; Table 1).

**TABLE 1:** Baseline patient characteristics.

	Total (*n* = 167)	Low‐FNG group (*n* = 59)	High‐FNG group (*n* = 95)	*p*
Sex				
Male	144 (86.2%)	53 (89.8%)	79 (83.2%)	0.250
Female	23 (13.8%)	6 (10.2%)	16 (16.8%)	
Age median (range)	70 (39–86)	72 (39–84)	69 (40–86)	0.384
Disease status				
Recurrent	97 (58.1%)	43 (72.9%)	45 (47.4%)	0.002
Metastatic	70 (41.9%)	16 (27.1%)	50 (52.6%)	
Line number of treatments				
1st	76 (45.5%)	26 (44.1%)	45 (47.4%)	0.399
2nd and beyond	91 (54.5%)	33 (55.9%)	50 (52.6%)	
Number of metastatic organs (mean ± SD)	3.5 ± 3.3	3.3 ± 3.1	3.7 ± 3.4	0.246

*Note:* The number of metastatic organs was defined as the total count of metastatic involvement across the following sites: the primary or locally recurrent tumor (0 or 1), bone metastasis (0 or 1), number of lung metastases, number of liver metastases, presence of regional and extra‐regional lymph node metastases in the cervical, thoracic, and abdominal regions (each scored as 0 or 1), brain metastasis (0 or 1), peritoneal dissemination (0 or 1), and other organ metastases including muscle or skin involvement (0 or 1).

Abbreviations: CRP, C‐reactive protein; FNG, fibrinogen.

**FIGURE 1:**
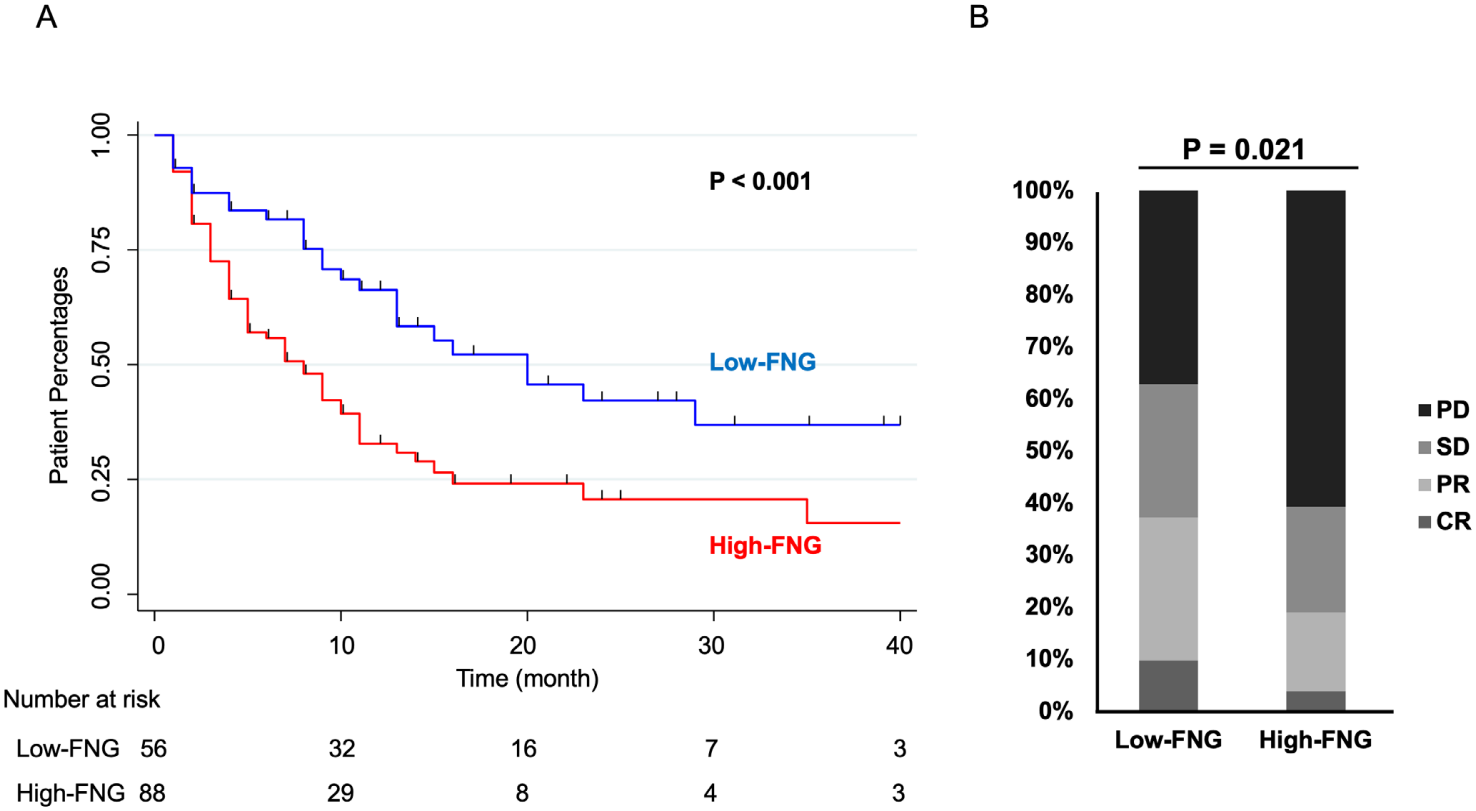
(A) Kaplan–Meier curves of overall survival (OS) for high‐ and low‐FNG groups. Red line: High‐FNG group; blue line: Low‐FNG group. (B) Immune checkpoint inhibitor (ICI) therapy response in patients with unresectable progressive or recurrent esophageal cancer. The high‐FNG group had significantly more patients with progressive disease (PD) than the low‐FNG group.

Survival analysis showed significantly shorter OS in the high‐FNG group compared to the low‐FNG group. The 3‐year OS was 36.9% for the low‐FNG group and 15.5% for the high‐FNG group (HR, 2.11; 95% CI, 1.33–3.37; *p* < 0.001) (Figure 1A). The high‐FNG group also had a significantly lower best overall response rate (PR + CR: 19.0% vs. 37.3%, *p* = 0.021, Figure 1B). Based on the observed data, with a hazard ratio of 2.11, total number of events of 94, distribution ratio of 7:11, and two‐sided significance level of 5%, the statistical power of the log‐rank test was 0.942, and a certain level of power was retained. In order to evaluate the prognostic value of FNG, we performed a multivariable analysis using age (≥ 65 years), sex, FNG level (> 390 mg/dL), the number of metastatic organs, and recurrent disease as covariates. As a result, a high FNG level (> 390 mg/dL) was demonstrated to be an independent prognostic factor (HR, 2.381; 95% CI, 1.455–3.896; *p* = 0.001; Table 2).

**TABLE 2:** Univariable and multivariable Cox regression analyses for overall survival.

	Univariate analysis			Multivariate analysis		
	HR	95% CI	*p*	HR	95% CI	*p*
Age (> 65 years old)	1.120	0.709–1.767	0.628	1.165	0.727–1.867	0.526
FNG (> 390 mg/dL)	2.114	1.326–3.371	0.002	2.381	1.455–3.896	0.001
Number of metastatic organs	1.017	0.957–1.080	0.588	1.025	0.961–1.093	0.452
Sex	1.206	0.604–2.407	0.595	1.396	0.688–2.834	0.355
Recurrent	1.037	0.676–1.589	0.868	1.393	0.855–2.271	0.183

*Note:* The number of metastatic organs was defined as the total count of metastatic involvement across the following sites: the primary or locally recurrent tumor (0 or 1), bone metastasis (0 or 1), number of lung metastases, number of liver metastases, presence of regional and extra‐regional lymph node metastases in the cervical, thoracic, and abdominal regions (each scored as 0 or 1), brain metastasis (0 or 1), peritoneal dissemination (0 or 1), and other organ metastases including muscle or skin involvement (0 or 1).

### 3.2 Efficacy of Chemo‐ICI vs. Dual‐ICI Therapy in High‐ vs. Low‐FNG Groups

We investigated whether first‐line chemo‐ or dual‐ICI therapy efficacy differed between high‐ and low‐FNG groups. We reviewed 59 previously treated patients with unresectable progression or recurrent ESCC who received these therapies as a first‐line treatment between January 2017 and March 2024. A total of 35 patients were in the high‐FNG group and 20 in the low‐FNG group. To directly assess tumor burden, we compared the number of metastatic organs between groups. Among patients with high FNG receiving first‐line chemo‐ICI or dual‐ICI therapy (*n* = 35), the number of metastatic organs was comparable (4.0 ± 3.0 vs. 3.3 ± 2.5, *p* = 0.232). No significant difference was found in patients with low FNG (4.5 ± 2.3 vs. 3.5 ± 2.0, *p* = 0.427; *n* = 20). Subsequently, we examined whether differences existed between the groups with respect to systemic inflammation. In the high‐FNG subgroup (*n* = 35), CRP levels were similar between the chemo‐ICI and dual‐ICI groups (2.70 ± 4.79 vs. 2.67 ± 2.59, *p* = 0.627). Likewise, in the low‐FNG subgroup, CRP levels did not differ significantly (1.12 ± 2.11 vs. 0.24 ± 0.27, *p* = 0.322). These findings suggest that, from the perspective of systemic inflammation, which closely reflects tumor burden, the two treatment groups were comparable (Table S1). Furthermore, we compared overall survival between the groups. Among the 55 patients with unresectable advanced or recurrent ESCC who received first‐line chemo‐ICI or dual‐ICI therapy, overall survival did not differ significantly between the groups (HR, 0.958; 95% CI, 0.41–2.22; *p* = 0.919), indicating no substantial differences in disease progression. Overall, tumor burden was found to be comparable between the two treatment groups.

**FIGURE 2:**
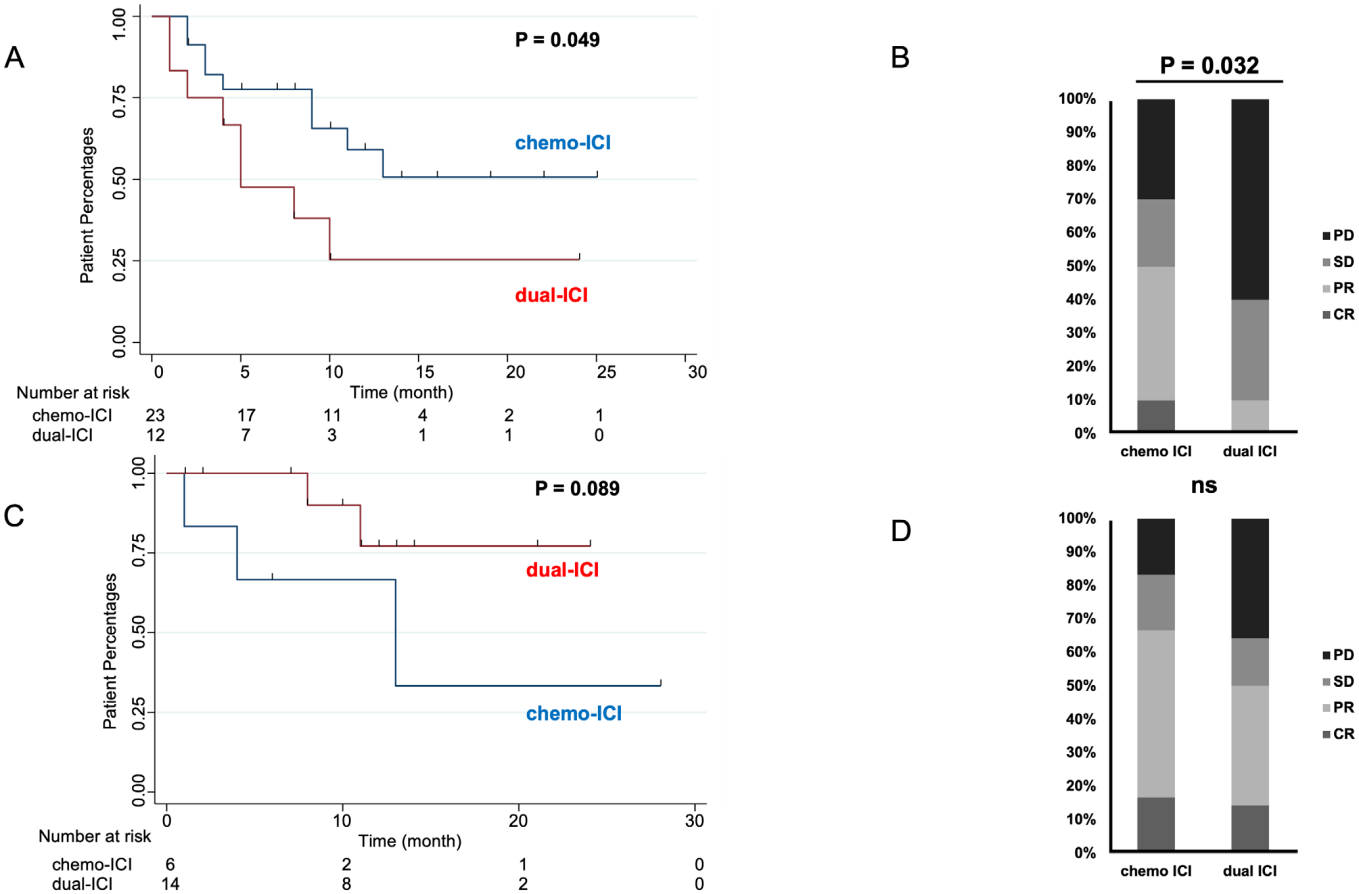
(A) Kaplan–Meier curves of overall survival for chemo‐ICI vs. dual‐ICI therapy in the high‐FNG group with unresectable progressive or recurrent ESCC as first‐line treatment. (B) First‐line ICI‐therapy response in high‐FNG group patients. The dual‐ICI group had significantly more patients with PD than the chemo‐ICI group. (C) Kaplan–Meier curves of overall survival for chemo‐ICI vs. dual‐ICI therapy in the low‐FNG group with unresectable progressive or recurrent ESCC as first‐line treatment. (D) First‐line ICI therapy response in low‐FNG group patients. The chemo‐ICI group had significantly more patients with PD than the dual‐ICI group.

In the high‐FNG group, chemo‐ICI therapy showed significantly longer OS than dual‐ICI therapy, with 2‐year OS rates of 50.7% and 25.4% (*p* = 0.049), respectively (Figure 2A). Chemo‐ICI also had a significantly higher best overall response rate (PR + CR 50.0% vs. 10.0%, *p* = 0.032, Figure 2B). Conversely, in the low‐FNG group, chemo‐ICI therapy trended toward shorter OS than dual‐ICI therapy, with 2‐year OS rates of 33.3% and 77.1% (HR, 4.28; 95% CI, 0.70–26.3; *p* = 0.089), respectively (Figure 2C). Best overall response rates did not differ significantly between chemo‐ICI and dual‐ICI in the low‐FNG group (PR + CR: 66.7% vs. 50.0%, *p* = 0.492) (Figure 2D).

### 3.3 Tumor‐Associated Neutrophils and Plasma FNG

Tumor‐associated neutrophils (TANs), a subset of neutrophils present within the tumor microenvironment (TME), are generally recognized to possess antitumor properties, including direct cytotoxicity and metastasis suppression [40]. Based on this, we hypothesized that TAN infiltration is elevated in the high‐FNG group. To investigate this, we used surgical specimens from 91 ESCC patients who underwent transthoracic esophagectomy at our institution between January 2014 and December 2022. The patient population demographics were: 86% male (*n* = 78), median age 67 years, with the following pStage distribution: I/II/III/IV, 0/19/62/10 (0.0/20.9/68.1/11.0%). The 3‐year OS and recurrence‐free survival rates were 40.1% and 23.6%, respectively.

**FIGURE 3:**
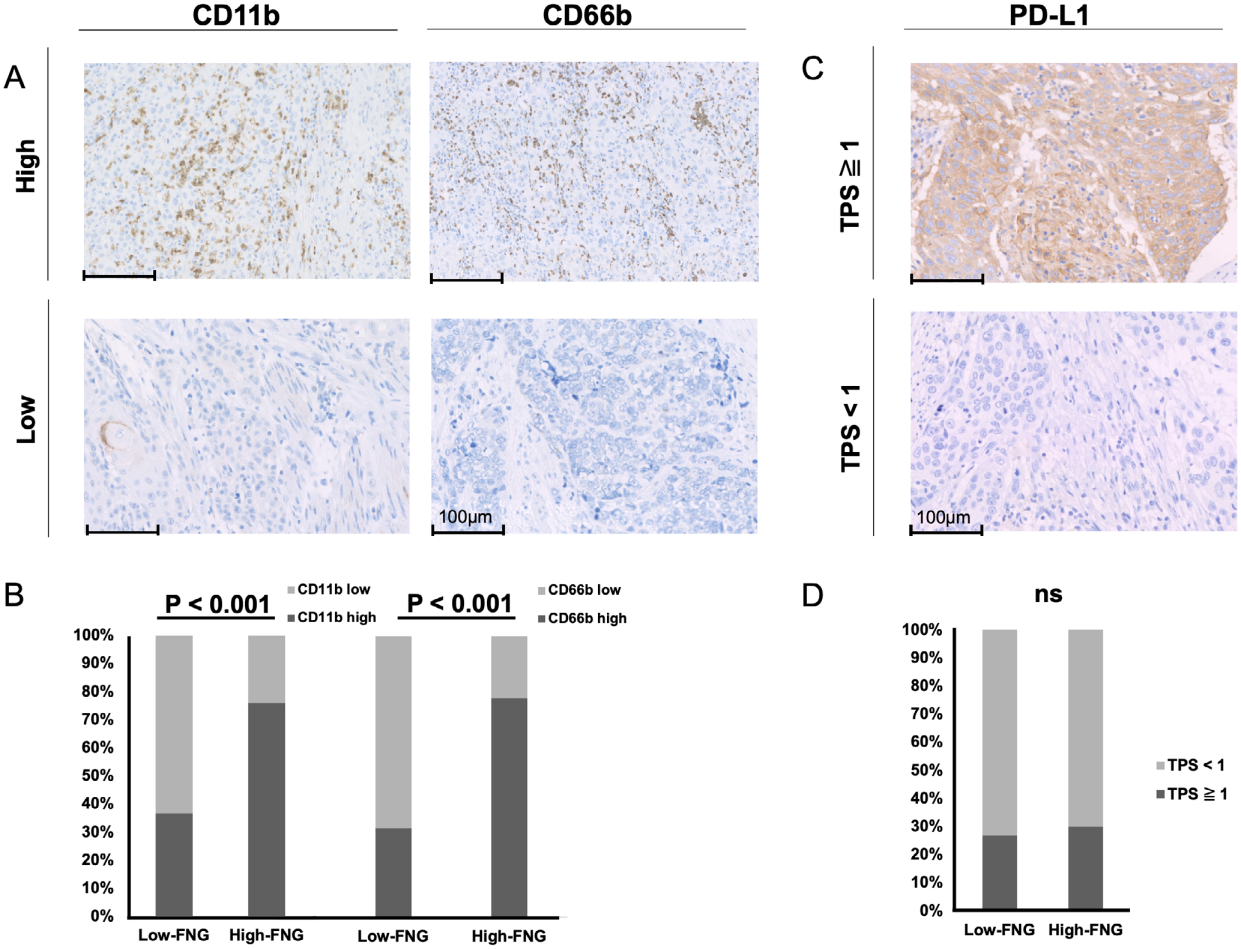
(A) Immunohistochemistry for CD11b and CD66b in ESCC tumors. Original magnification, 400×; scale bar, 100 μm. (B) Percentage of CD11b‐ and CD66b‐stained cells. (C) Immunohistochemistry for PD‐L1 in ESCC tumors. (D) Percentage of PD‐L1‐positive stained cells.

To evaluate neutrophil infiltration, we conducted immunohistochemical staining using CD11b and CD66b, common neutrophil markers (Figure 3A). The proportion of stained cells was used to score neutrophil infiltration in tumor cells, and samples were classified into high and low groups based on a 20% cutoff. The staining results for CD11b and CD66b were highly consistent, revealing a significantly higher presence of neutrophils in the high‐FNG group (Table S2, Figure 3B).

### 3.4 PD‐L1 Expression and Plasma FNG

To investigate the potential link between FNG levels and PD‐L1 expression, a key biomarker in esophageal cancer, we analyzed surgical specimens from 91 ESCC patients (January 2014–December 2022). PD‐L1/28‐8 immunohistochemical staining was performed (Figure 3C), and samples were classified into high and low TPS groups using a 1% cutoff. While approximately 30% of specimens were TPS‐high, no correlation was found between FNG levels, neutrophil infiltration in the primary tumor, and PD‐L1 expression (Table S2 and Table 3, Figure 3D).

**TABLE 3:** Correlation between PD‐L1 expression, pathological stage and tumor‐infiltrating neutrophil counts.

	TPS < 1 group (*n* = 65)	TPS ≧ 1 group (*n* = 26)	*p*
pStage (UICC 8th)			
I	0 (0.0%)	0 (0.0%)	0.935
II	13 (20.0%)	6 (23.1%)	
III	45 (69.2%)	17 (65.4%)	
IV	7 (10.8%)	3 (11.5%)	
CD11b			
High	37 (56.9%)	16 (61.5%)	0.687
Low	28 (43.1%)	10 (38.5%)	
CD66b			
High	35 (53.8%)	17 (65.4%)	0.315
Low	30 (46.2%)	9 (34.6%)	

### 3.5 Genomic Profile and Plasma FNG Levels

**FIGURE 4:**
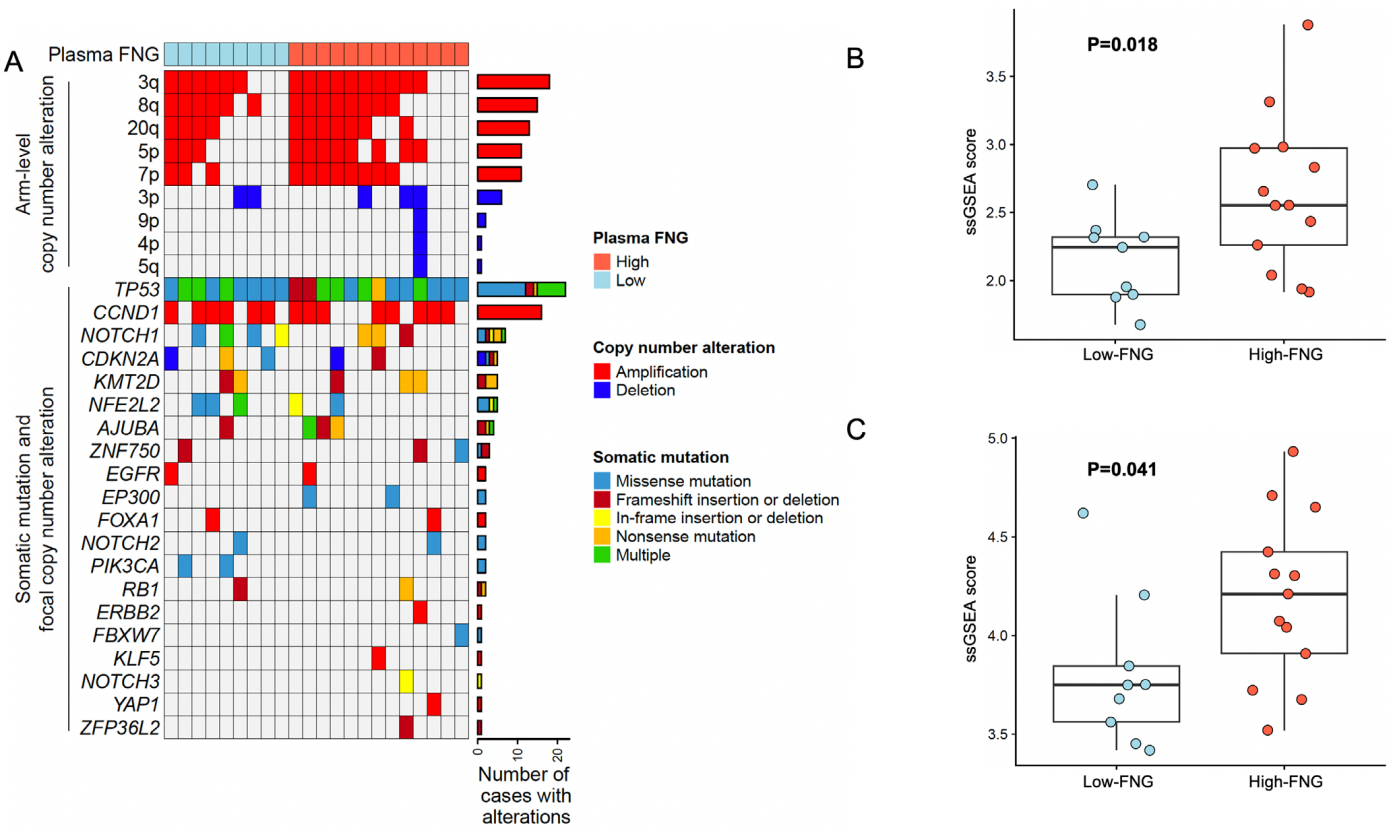
(A) Heatmap depicting copy number alterations and somatic mutations in key genes within ESCC tumors, stratified by plasma FNG levels. Red: Amplifications; blue: Deletion; color‐coded mutation types. The number of cases exhibiting each alteration is displayed. No significant differences were observed between high and low‐FNG groups, suggesting that genetic alterations do not drive elevated plasma FNG levels. (B) Boxplot illustrating ssGSEA scores for the regulation of neutrophil activation in ESCC tumors, stratified according to plasma FNG levels. Tumors with high‐FNG levels showed a statistically significant increase in neutrophil activation scores compared to tumors with low‐FNG levels (*p* = 0.018). (C) Boxplot illustrating ssGSEA scores for neutrophil extravasation in ESCC tumors, stratified according to plasma FNG levels. Tumors with high‐FNG levels showed a statistically significant increase in neutrophil extravasation scores compared to tumors with low‐FNG levels (*p* = 0.041).

To investigate whether tumor genomic alterations correlate with FNG levels, we performed WES on esophagectomy specimens. A comparative analysis of patients with high (*n* = 13) and low (*n* = 9) preoperative FNG levels showed no significant differences in their genomic profiles. The frequency of key driver mutations, such as TP53, CCND1, NOTCH1, CDKN2A, and PIK3CA, was similar between the groups (Figure 4A). Furthermore, we found no enrichment of chromosomal arm‐level or focal CNAs, indicating that plasma FNG levels do not reflect underlying genomic changes in esophageal cancer.

Using ssGSEA, RNA sequencing analysis quantified enrichment scores for neutrophil activation and extravasation gene sets from MSigDB. We found a striking upregulation of neutrophil‐related pathways in the high‐FNG group. Specifically, the regulation of the neutrophil activation gene set showed a markedly higher enrichment score in patients with high‐FNG levels (*p* = 0.018; Figure 4B), and the neutrophil extravasation gene set was also significantly enriched in this group (*p* = 0.041; Figure 4C).

## 4 Discussion

Utilizing a multicenter design, we investigated and confirmed the predictive value of plasma FNG levels for ICI outcomes in a large cohort of ESCC patients. Our findings revealed that elevated plasma FNG levels are associated with a significant reduction in ICI response. Specifically, for first‐line treatment, chemo‐ICI therapy demonstrated superior efficacy, including prolonged OS, compared to dual‐ICI therapy in patients with high FNG. Through comprehensive immunohistochemical profiling, we observed a strong association between high‐FNG levels and increased neutrophil infiltration within primary tumors, a result validated by RNA sequencing. However, no significant differences were linked to FNG levels. This study provides crucial evidence for the utility of FNG as a biomarker to inform first‐line treatment decisions between chemo‐ and dual‐ICI in ESCC.

By incorporating multicenter clinical data, this study expands upon previous investigations, such as Hoshino et al. to provide a more comprehensive evaluation of the correlation between elevated FNG levels and ICI efficacy. We acknowledge the established mechanisms by which (i) PD‐1 inhibitors (nivolumab and pembrolizumab) can enhance cytotoxic T‐cell activity of tumor cells [26, 41], and (ii) ipilimumab, an anti‐CTLA‐4 antibody, blocks inhibitory regulation in activated T cells, thereby both promoting antigen‐specific T‐cell activation and also enhancing the cytotoxic activity of tumor cells [42]. Our findings indicate that high‐FNG levels, potentially reflecting an immunosuppressive TME with high inflammation, are associated with significantly lower ICI response rates and poorer survival in ESCC. This study provides strong evidence for the clinical utility of FNG as a simple and practical biomarker for predicting ICI efficacy.

Our study's initial analysis included a range of ICI regimens across different treatment settings. Based on the KEYNOTE‐590 and CheckMate 648 trials, chemo‐ or dual‐ICI therapy is now recommended by the Japanese Treatment Guidelines 2022 as first‐line treatments for unresectable advanced or recurrent ESCC [23]. Within this standard of care, we found that high‐FNG levels predicted poor responses to dual‐ICI therapy but better outcomes with chemo‐ICI. This result suggests that in the highly inflammatory, immunosuppressive TMEs associated with high FNG, ICIs alone may be insufficient, and cytotoxic agents are needed. In contrast, although not statistically significant due to sample size, dual‐ICI therapy tended to result in better survival in low‐FNG patients. This indicates that in less inflamed, immunologically active TMEs, dual‐ICI therapy may be preferable, as cytotoxic agents could negatively impact critical immune cells.

Our immunohistochemical analyses revealed a significant association between elevated plasma FNG levels and neutrophil infiltration in primary tumors, confirming that FNG reflects inflammatory activity in the TME. Though FNG is primarily produced by the liver in response to cytokines like IL‐6 and IL‐1β, our data suggest that tumor‐derived cytokines also contribute to elevated FNG. Importantly, FNG levels and neutrophil infiltration did not correlate with PD‐L1 expression, indicating FNG reflects distinct biological pathways. Studies on TANs in laryngeal squamous cell carcinoma have shown an inverse relationship between TANs and T cells, impairing “CD8+” T‐cell function and promoting immune evasion and tumor progression [43, 44, 45]. Consequently, we found high‐FNG levels correlated with TAN accumulation and an immunosuppressive TME. Therefore, rather than directly regulating the TME or the PD‐L1/PD‐1 signaling pathway, FNG should be considered a useful biomarker that reflects the real‐time immunological tumor environment in the primary lesion. This study is the first to establish a strong link between plasma FNG levels and TAN accumulation in ESCC primary tumors.

Transcriptomic analyses further validated our results, showing significant enrichment of neutrophil activation and extravasation pathways in tumors from patients with high FNG. ssGSEA revealed a clear upregulation of neutrophil‐related gene signatures, demonstrating that TAN accumulation is actively regulated within the TME, not just a systemic effect. Neutrophils are increasingly understood as key drivers of immune suppression, promoting tumor progression [40]. The strong link between elevated FNG and neutrophil‐driven inflammation reinforces the concept that TANs contribute to an immunosuppressive TME in ESCC, particularly by hindering T‐ and NK‐cell antitumor immunity. Given neutrophils' significant role in shaping tumor immunity, plasma FNG levels may serve as a clinically useful surrogate marker for neutrophil‐mediated immune suppression and ICI resistance. Targeting neutrophil pathways offers a promising therapeutic strategy to overcome ICI resistance, necessitating further research into the FNG‐neutrophil interaction and the potential of neutrophil‐modulating therapies.

This study is subject to several limitations. First, the retrospective nature of the study design may introduce selection bias. Furthermore, variations in treatment eligibility criteria and therapeutic approaches throughout the study period may have influenced the observed results. To mitigate selection bias, consecutive patients were selected for inclusion. Second, the sample size for cases involving first‐line ICI combination regimens was limited to cases treated after ICI became reimbursable in Japan, and because case accrual was restricted to institutions that routinely measure serum FNG as part of ESCC surveillance, it was challenging to accumulate a sufficient sample size. Therefore, this investigation was conducted as a multicenter collaborative study to maximize case accrual. Third, evaluating plasma FNG levels both before and after treatment would allow for a more detailed assessment. However, because this was a retrospective study, posttreatment plasma FNG levels were not available for many patients, making comprehensive data collection difficult. A prospective study is planned to evaluate posttreatment changes in plasma FNG during the clinical course. Taken together, these findings necessitate validation through larger, prospective cohort studies.

In conclusion, this study demonstrates that high plasma FNG levels are associated with worse outcomes and diminished ICI efficacy in ESCC patients. Plasma FNG levels may serve as a key biomarker to guide the choice of first‐line ICI‐based combination therapies for unresectable advanced or recurrent ESCC. Furthermore, the strong correlation between FNG levels and TAN infiltration in primary tumors suggests FNG's role as an indicator of TME inflammation. Future molecular studies are needed to confirm these clinical findings and investigate the mechanisms regulating TAN infiltration within the TME.

## Author Contributions

**Keiso Ho:** investigation, methodology, validation, visualization, writing – original draft, writing – review and editing. **Satoru Matsuda:** conceptualization, investigation, methodology, validation, visualization, writing – original draft, writing – review and editing. **Eisuke Booka:** writing – data collection. **Wataru Soneda:** writing – data collection. **Jun Okui:** writing – data analysis. **Shota Hoshino:** writing, review, and editing. **Masashi Takeuchi:** writing, review and editing. **Kazumasa Fukuda:** writing, review and editing. **Sara Horie:** writing – data analysis. **Yuki Saito:** writing – data analysis. **Yasunori Kogure:** writing – data analysis. **Hirofumi Kawakubo:** writing, review, and editing. **Kensuke Hara:** writing – data analysis. **Hajime Okita:** writing – data collection. **Keisuke Kataoka:** project administration, supervision, writing – review and editing. **Shigeki Sekine:** project administration, supervision, writing – review and editing. **Hiroya Takeuchi:** project administration, supervision, writing – review and editing. **Yuko Kitagawa:** project administration, supervision, writing – review and editing.

## Funding

This work was supported by the Takeda Science Foundation and JSPS KAKENHI Grant Number JP23K15504.

## Ethics Statement

The study protocol was approved by the Ethics Committee, Keio University Hospital (Approval Number: 20231201) and Hamamatsu University Hospital (Approval Number: 91‐244). The study was performed in accordance with the Declaration of Helsinki.

## Consent

The authors have nothing to report.

## Conflicts of Interest

Prof. Yuko Kitagawa is an Editorial Board member of Cancer Science. Prof. Kitagawa reports personal fees from MSD, personal fees from Bristol‐Myers Squibb K.K., personal fees from ONO PHARMACEUTICAL CO. LTD., personal fees from Nippon Kayaku Co. Ltd., grants and personal fees from TAIHO PHARMACEUTICAL CO. LTD, grants and personal fees from Eisai Co. Ltd., grants and personal fees from Kyowa Kirin Co. Ltd., outside the submitted work. Other authors do not have a conflicts of interest.

## Supporting information


**Table S1:** Baseline characteristics of patients receiving chemo‐ICI or dual‐ICI therapy as first‐line therapy.
**Table S2:** Association between clinicopathological factors, neutrophil markers, PD‐L1 expression, and plasma FNG levels.

## Data Availability

The data that support the findings of this study are available on request from the corresponding author. The data are not publicly available due to privacy or ethical restrictions.
