# Supplementary material for: Plasma Fibrinogen Predicts Response to Immune Checkpoint Inhibitor by Inflammatory Tumor Microenvironment in Esophageal Cancer
Source: Cancer Med. 2026 Jan 18;15(1):e71548. doi: 10.1002/cam4.71548 (PMC12812519; doi:10.1002/cam4.71548)
Supplement: Supplementary file 1 — Table S1: Baseline characteristics of patients receiving chemo‐ICI or dual‐ICI therapy as first‐line therapy. Table S2: Association between clinicopathological factors, neutrophil markers, PD‐L1 expression, and plasma FNG levels. [file CAM4-15-e71548-s001.docx]

**Supplementary Table 1.** Baseline characteristics of patients receiving chemo-ICI or dual-ICI therapy as first-line therapy

| High-FNG group | | | | |
| --- | --- | --- | --- | --- |
|  | Total  (n = 35) | Chemo-ICI  (n = 23) | Dual-ICI  (n = 12) | p value |
| Sex  Male  Female | 29 (82.9%)  6 (17.1%) | 17 (73.9%)  6 (26.1%) | 12 (100%)  0 (0.0%) | 0.052 |
| Age, median (range) | 72 (40–86) | 72 (40–83) | 75.5 (45–86) | 0.088 |
| Disease status  Recurrent  Metastatic | 20 (57.1%)  15 (42.9%) | 17 (73.9%)  6 (26.1%) | 3 (25.0%)  9 (75.0%) | 0.006 |
| CRP (mean ± SD) | 2.69 ± 4.12 | 2.70 ± 4.79 | 2.67 ± 2.59 | 0.627 |
| Number of metastatic organs (mean ± SD) | 3.8 ± 2.8 | 4.0 ± 3.0 | 3.3 ± 2.5 | 0.232 |
| Low-FNG group | | | | |
|  | Total  (n = 20) | Chemo-ICI  (n = 6) | Dual-ICI  (n = 14) | p value |
| Sex  Male  Female | 18 (90.0%)  2 (10.0%) | 6 (100%)  0 (0.0%) | 12 (85.7%)  2 (14.3%) | 0.329 |
| Age median (range) | 65 (39–82) | 57 (47–76) | 67.5 (39–82) | 0.247 |
| Disease status  Recurrent  Metastatic | 5 (25.0%)  15 (75.0%) | 2 (33.3%)  4 (66.7%) | 3 (21.4%)  11 (78.6%) | 0.573 |
| CRP (mean ± SD) | 0.50 ± 1.18 | 1.12 ± 2.11 | 0.24 ± 0.27 | 0.322 |
| Number of metastatic organs (mean ± SD) | 3.8 ± 2.0 | 4.5 ± 2.3 | 3.5 ± 2.0 | 0.427 |

The number of metastatic organs was defined as the total count of metastatic involvement across the following sites: the primary or locally recurrent tumor (0 or 1), bone metastasis (0 or 1), number of lung metastases, number of liver metastases, presence of regional and extra-regional lymph node metastases in the cervical, thoracic, and abdominal regions (each scored as 0 or 1), brain metastasis (0 or 1), peritoneal dissemination (0 or 1), and other organ metastases including muscle or skin involvement (0 or 1).

**Supplementary table 2.** Association between clinicopathological factors, neutrophil markers, PD-L1 expression, and plasma FNG levels

|  | Low-FNG group  (n = 41) | High-FNG group  (n = 50) | p values |
| --- | --- | --- | --- |
| Sex  Male  Female | 38 (92.7%)  3 (7.3%) | 40 (80.0%)  10 (20.0%) | 0.085 |
| Age median (range) | 67 (36–82) | 69 (48–82) | 0.129 |
| pStage (UICC 8th)  I  II  III  IV | 0 (0.0%)  6 (14.6%)  31 (75.6%)  4 (9.8%) | 0 (0.0%)  13 (26.0%)  31 (62.0%)  6 (12.0%) | 0.348 |
| CD11b  high  low | 15 (36.6%)  26 (63.4%) | 38 (76.0%)  12 (24.0%) | <0.001 |
| CD66b  high  low | 13 (31.7%)  28 (68.3%) | 39 (78.0%)  11 (22.0%) | <0.001 |
| TPS  ≧1  <1 | 11 (26.8%)  30 (73.2%) | 15 (30.0%)  35 (70.0%) | 0.739 |

TPS, tumor proportion score.
